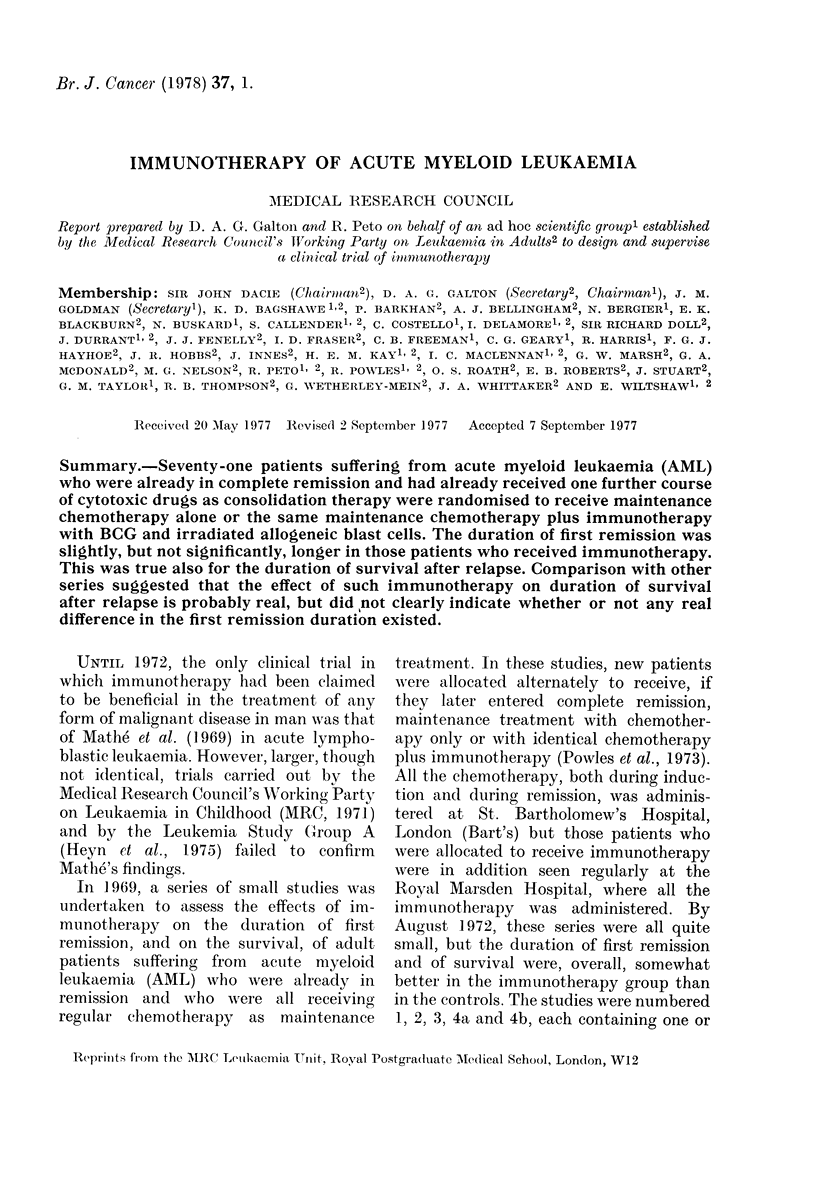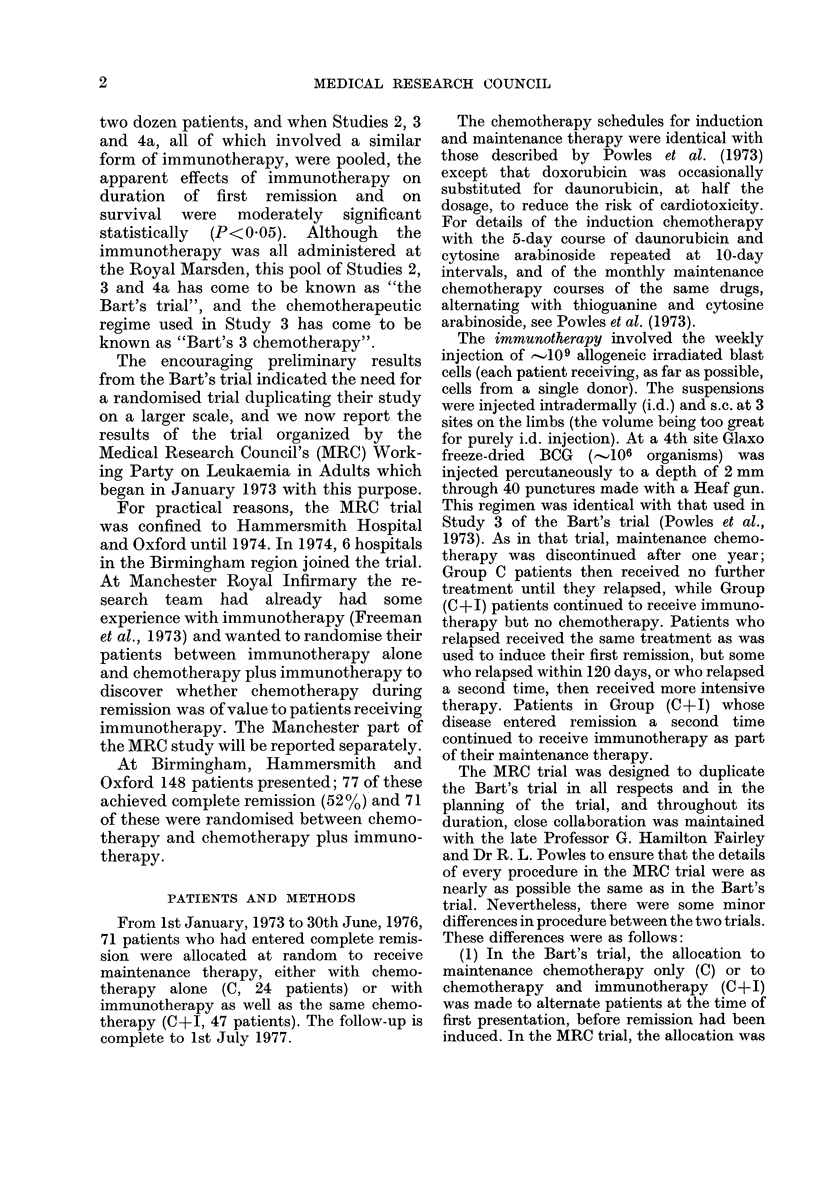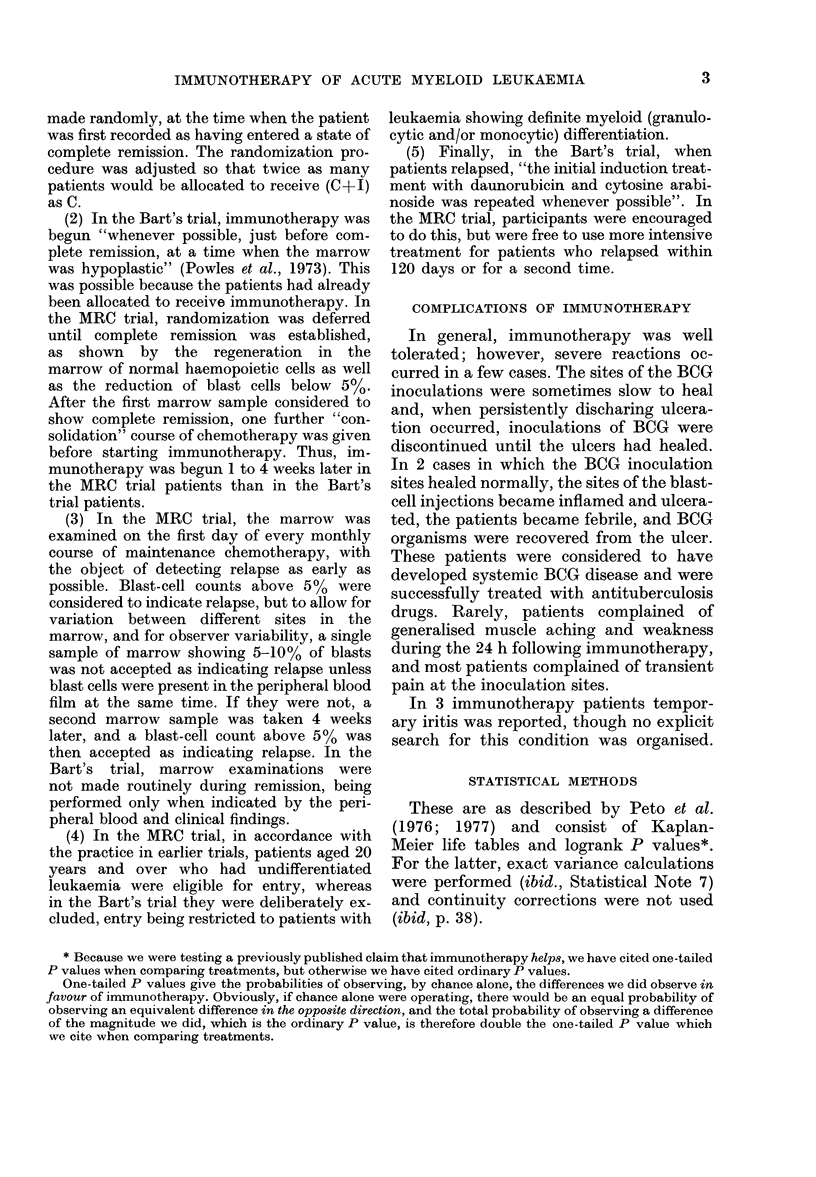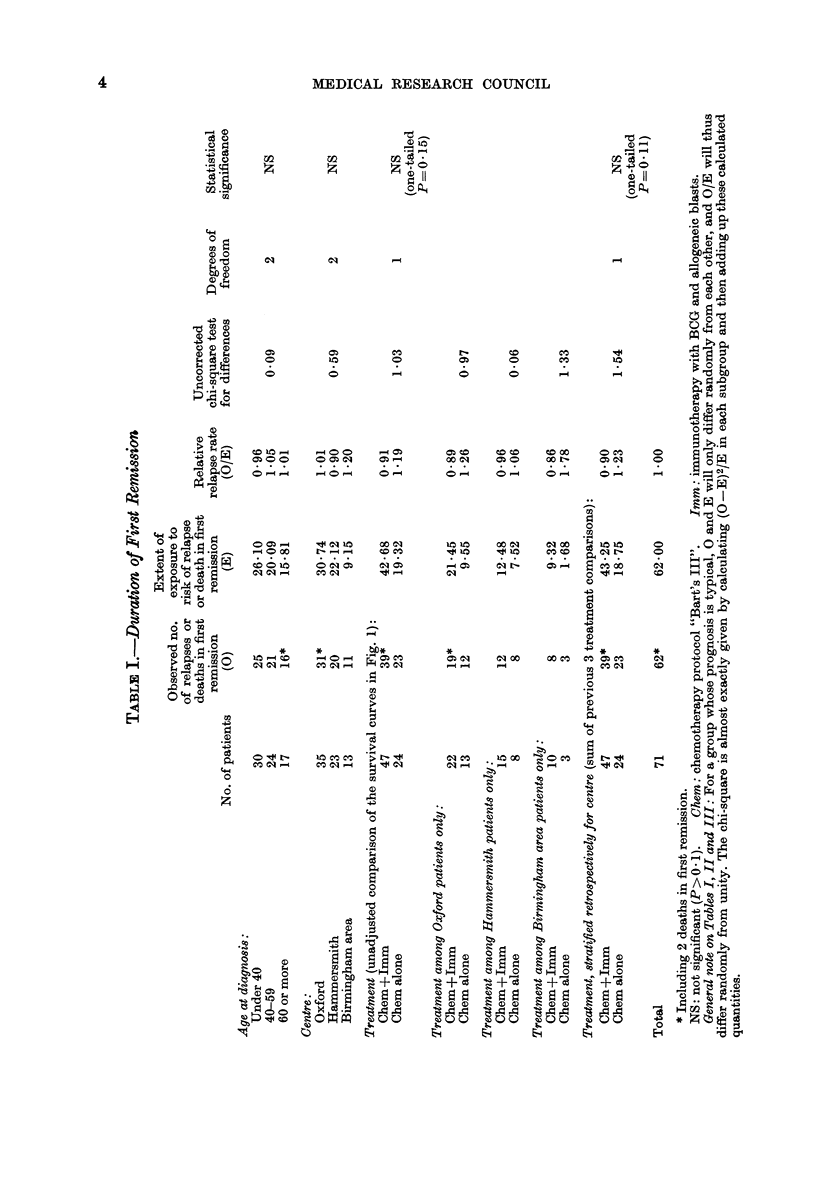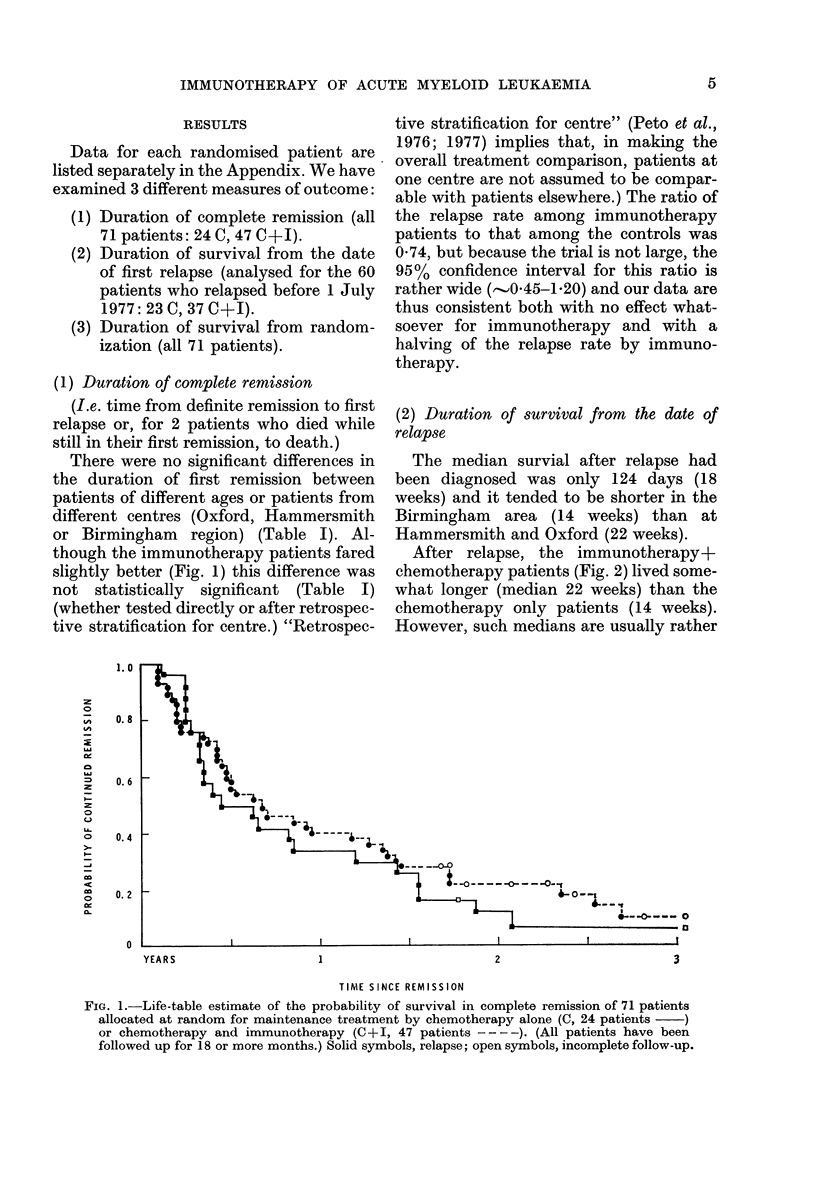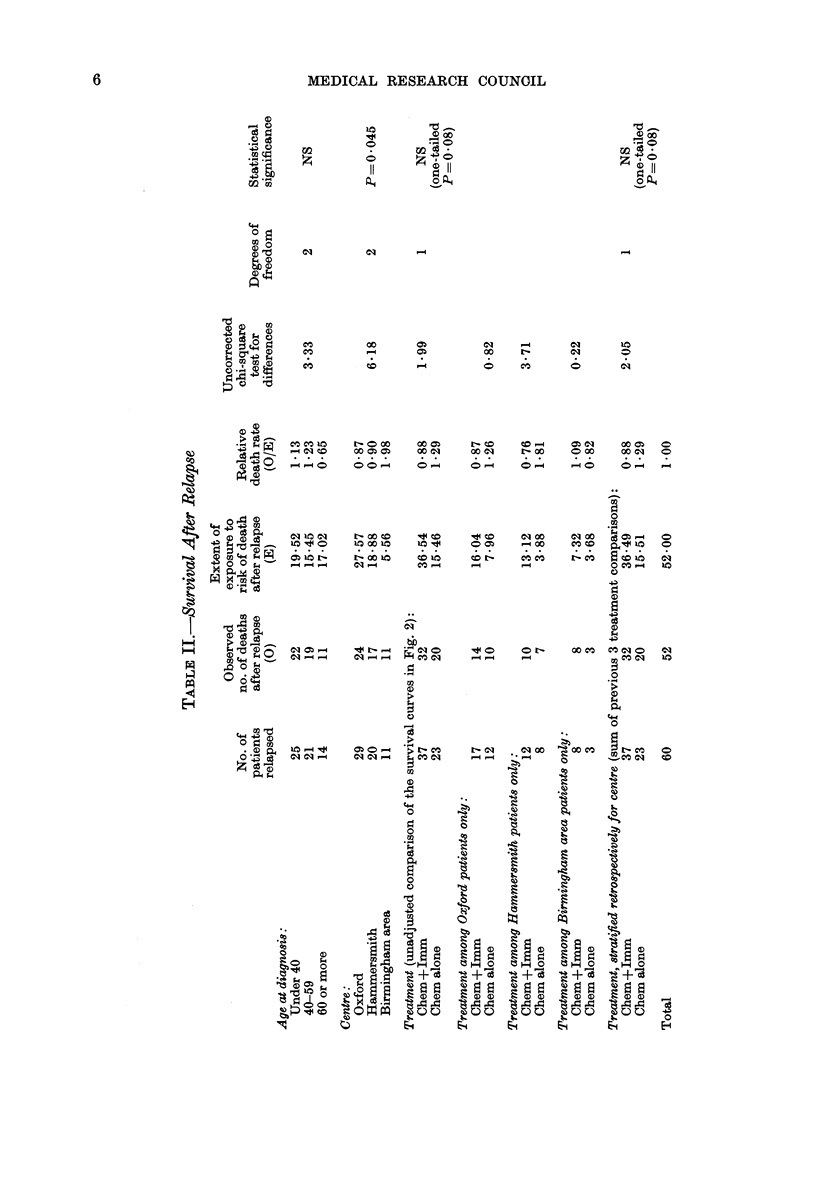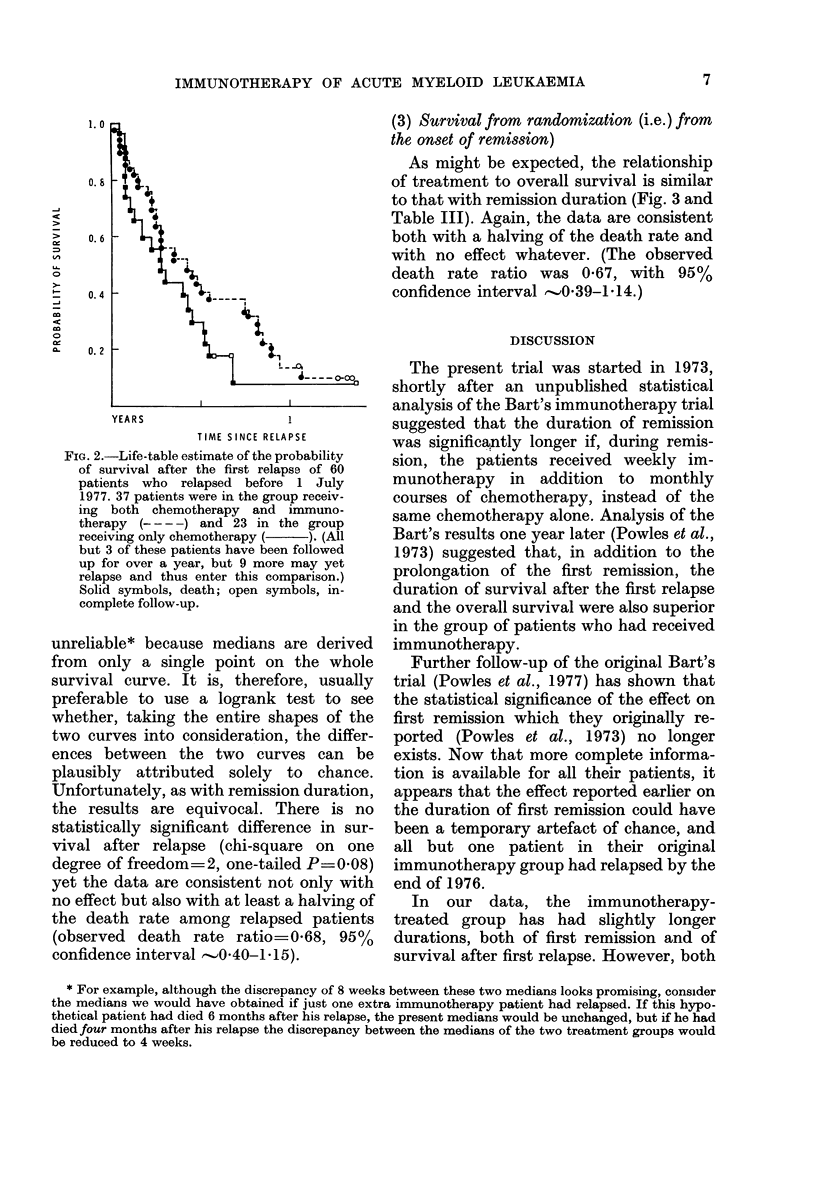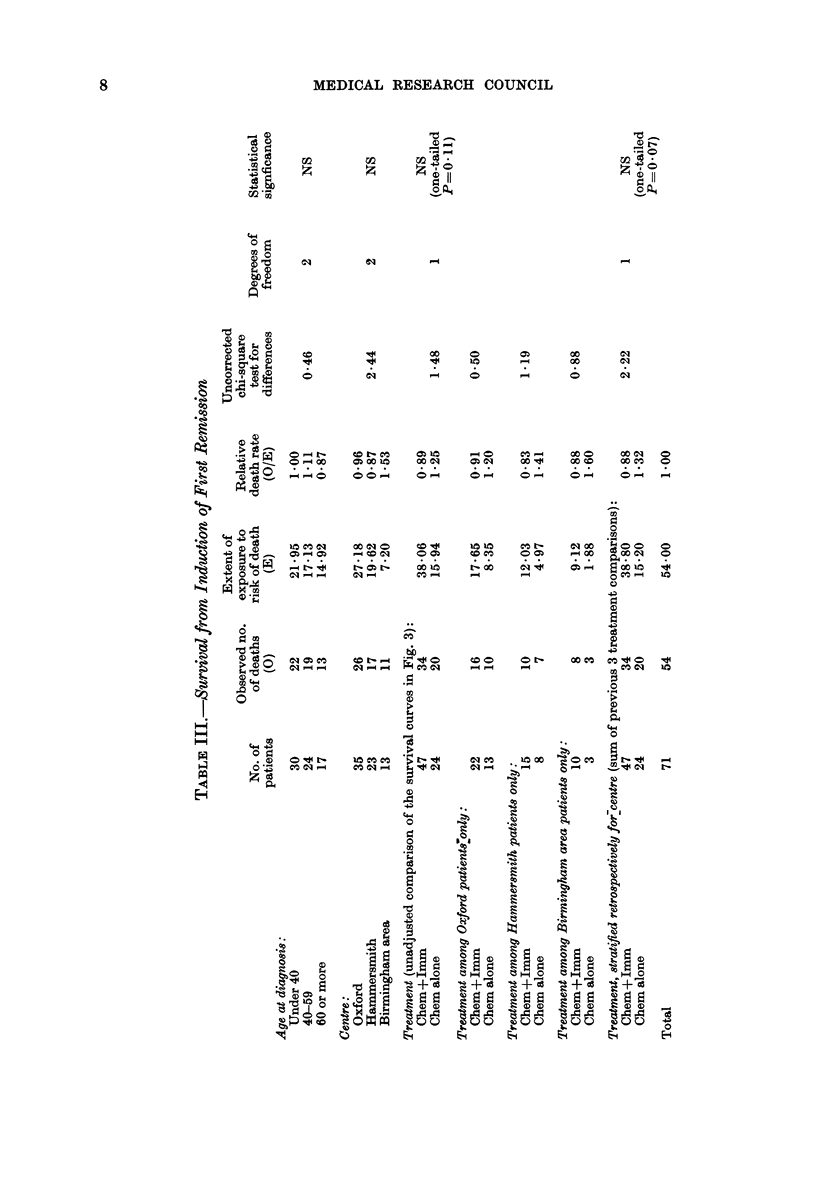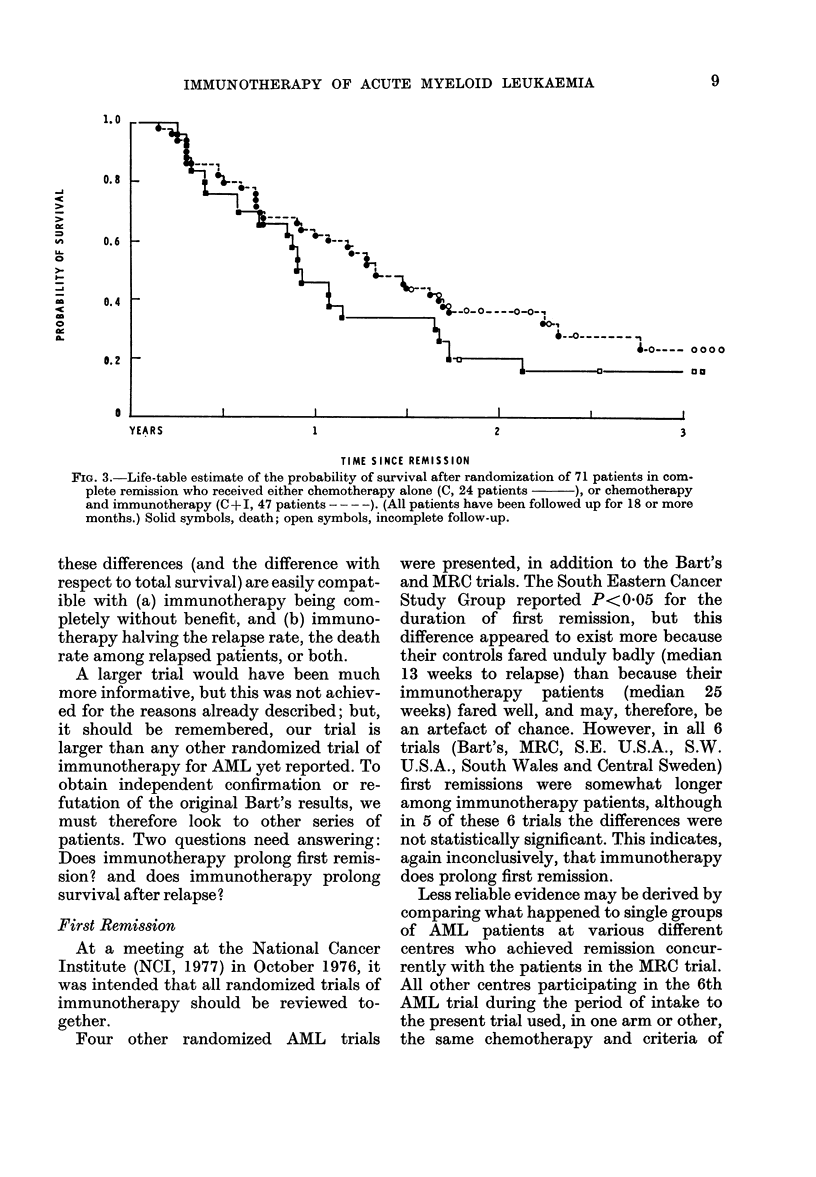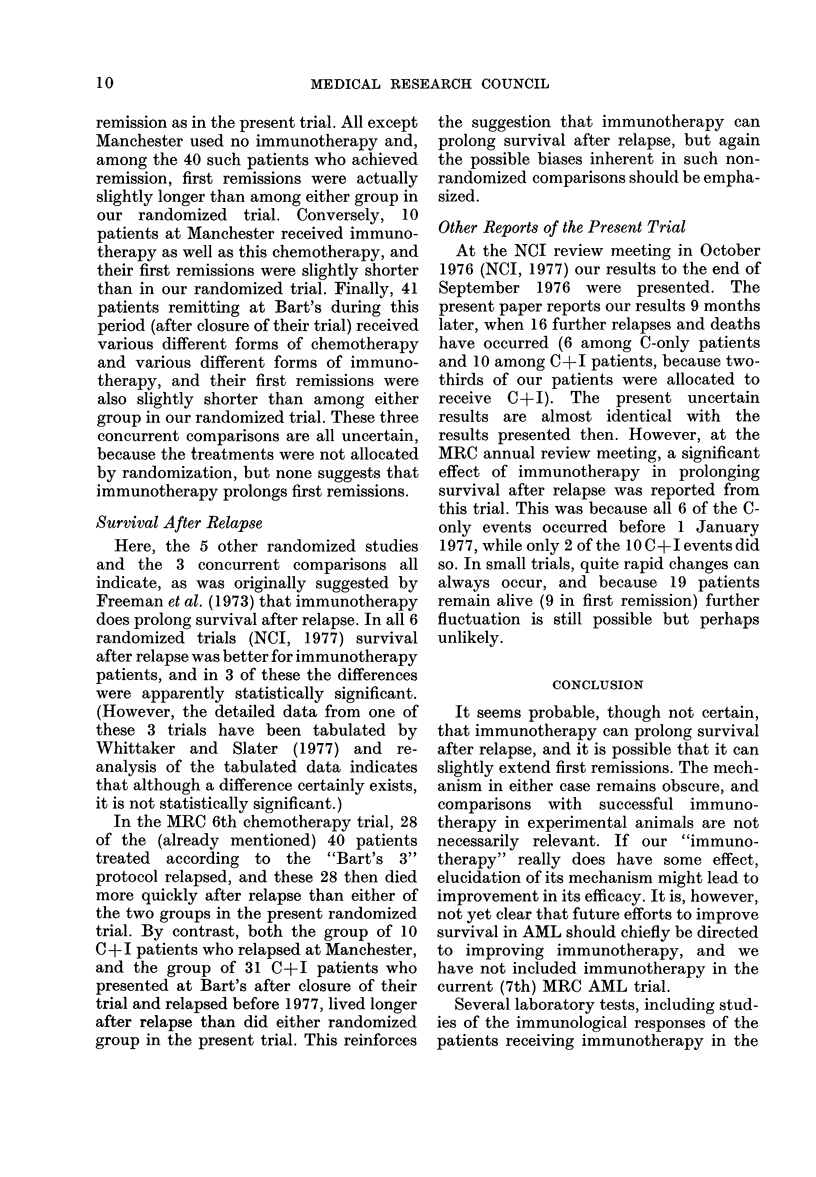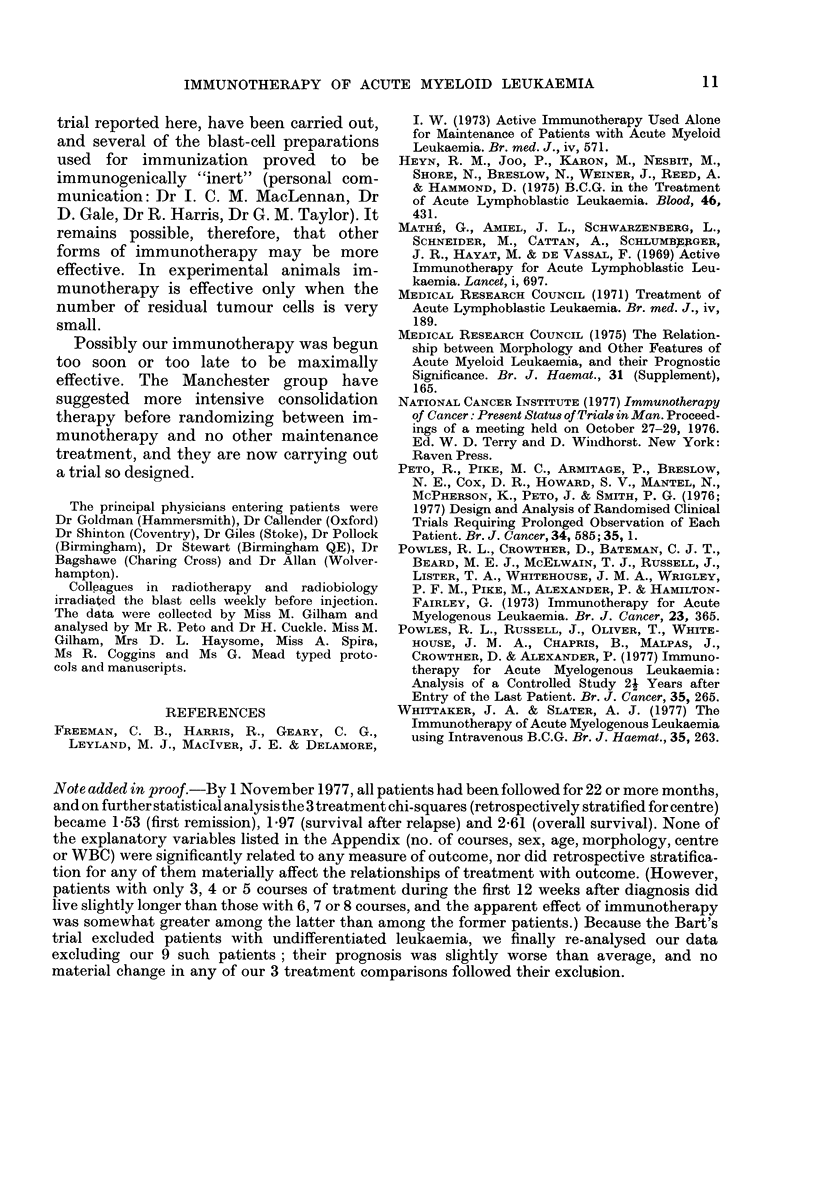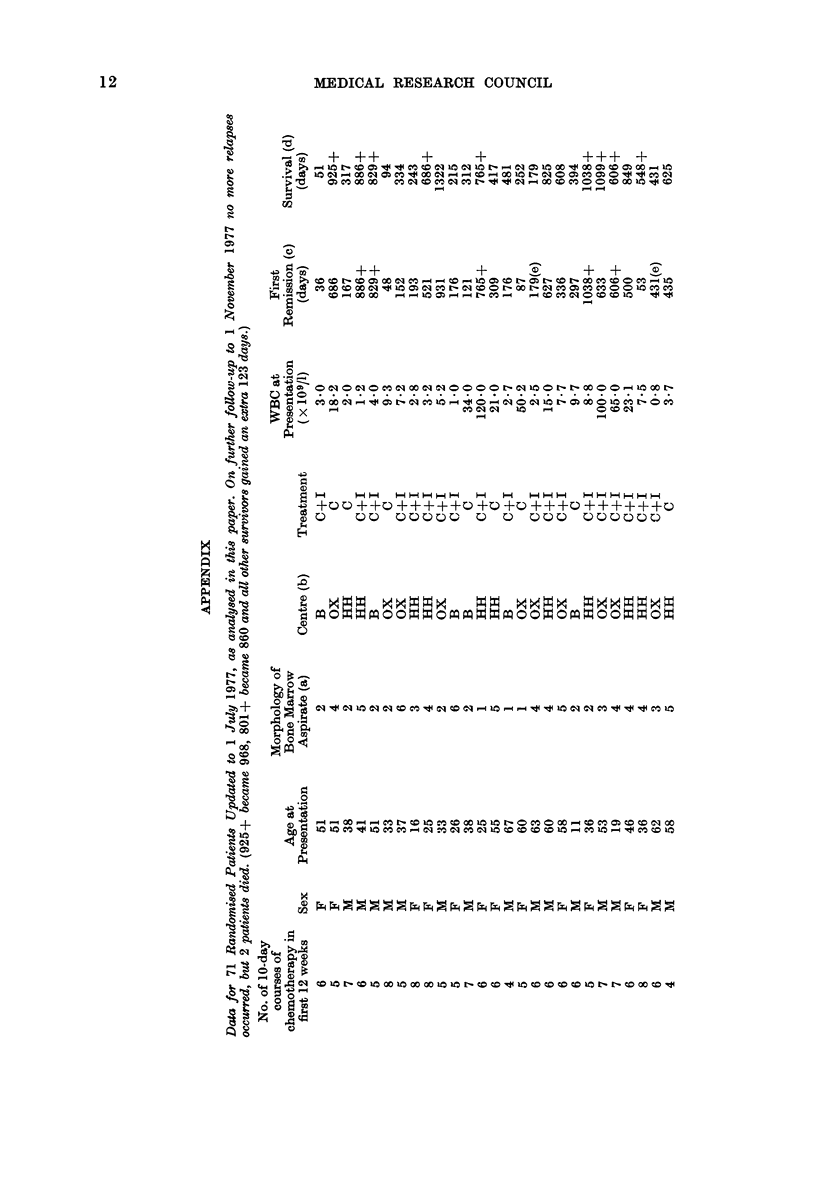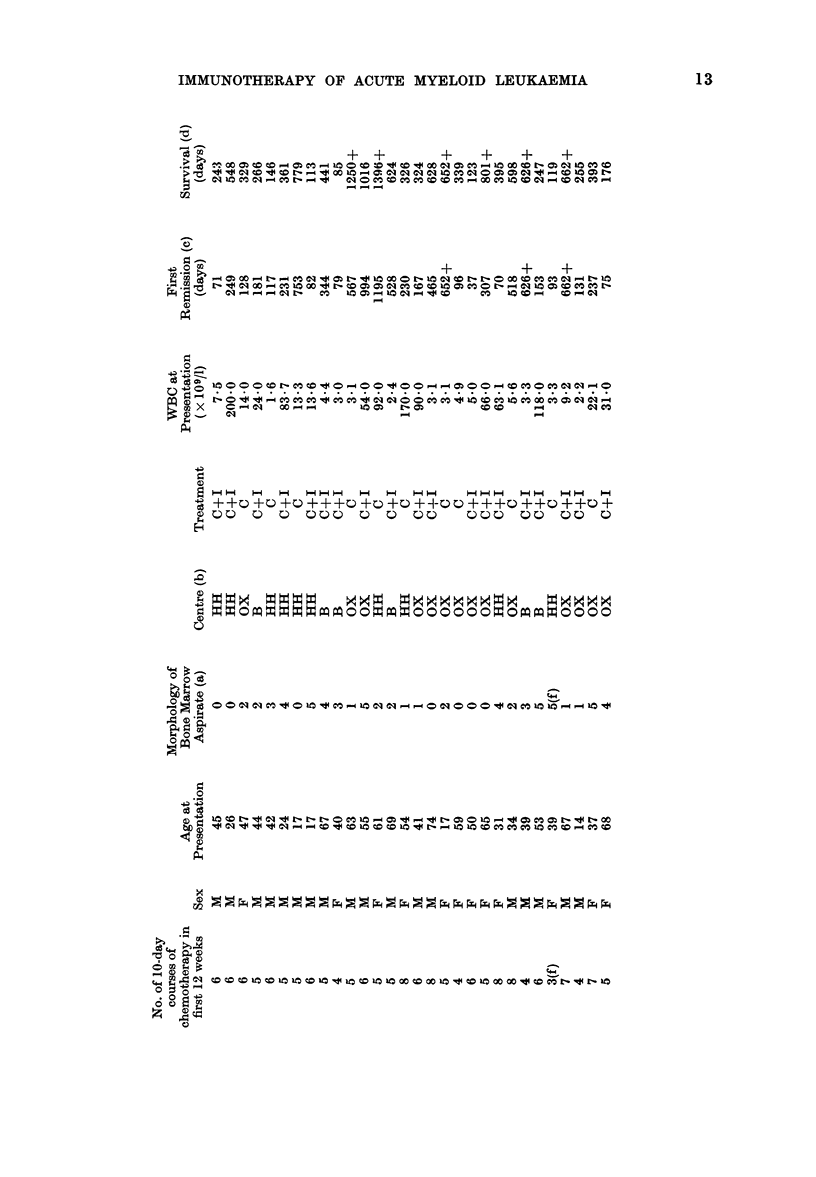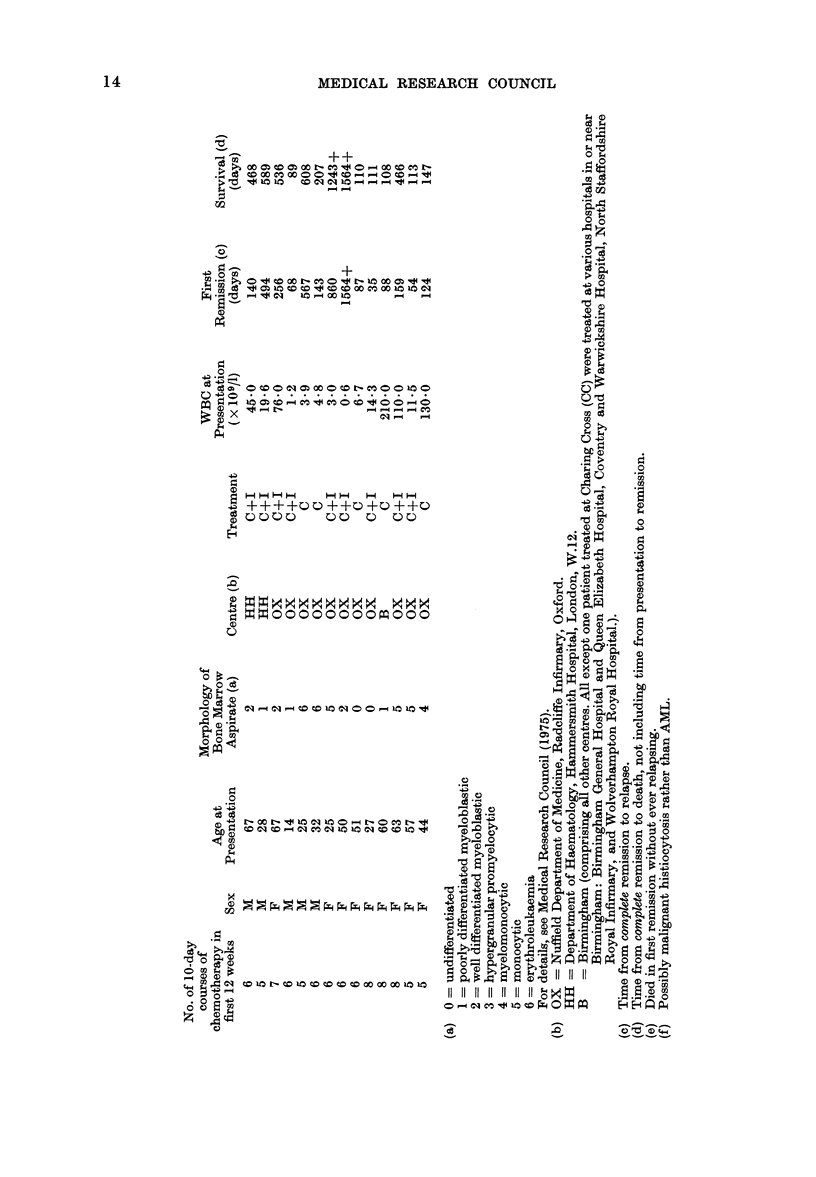# Immunotherapy of acute myeloid leukaemia. Medical Research Council.

**DOI:** 10.1038/bjc.1978.1

**Published:** 1978-01

**Authors:** 

## Abstract

Seventy-one patients suffering from acute myeloid leukaemia (AML) who were already in complete remission and had already received one further course of cytotoxic drugs as consolidation therapy were randomised to receive maintenance chemotherapy alone or the same maintenance chemotherapy plus immunotherapy with BCG and irradiated allogeneic blast cells. The duration of first remission was slightly, but not significantly, longer in those patients who received immunotherapy. This was true also for the duration of survival after relapse. Comparison with other series suggested that the effect of such immunotherapy on duration of survival after relapse is probably real, but did not clearly indicate whether or not any real difference in the first remission duration existed.


*Br. J. Cancer* (1978) **37**, 1.

### IMMUNOTHERAPY OF ACUTE MYELOID LEUKAEMIA

MEDICAL RESEARCH COUNCIL

*Report prepared by* D. A. G. Galton *and* R. Peto *on behalf of an* ad hoc *scientific group*[1] *established by the Medical Research Council's Working Party on Leukaemia in Adults*[2] *to design and supervise a clinical trial of immunotherapy*

**Membership:** SIR JOHN DACIE (*Chairman*[2]), D. A. G. GALTON (*Secretary*[2], *Chairman*[1]), J. M. GOLDMAN (*Secretary*[1]), K. D. BAGSHAWE[1,2], P. BARKHAN[2], A. J. BELLINGHAM[2], N. BERGIER[1], E. K. BLACKBURN[2], N. BUSKARD[1], S. CALLENDER[1,2], C. COSTELLO[1], I. DELAMORE[1,2], SIR RICHARD DOLL[2], J. DURRANT[1,2], J. J. FENELLY[2], I. D. FRASER[2], C. B. FREEMAN[1], C. G. GEARY[1], R. HARRIS[1], F. G. J. HAYHOE[2], J. R. HOBBS[2], J. INNES[2], H. E. M. KAY[1,2], I. C. MACLENNAN[1,2], G. W. MARSH[2], G. A. MCDONALD[2], M. G. NELSON[2], R. PETO[1,2], R. POWLES[1,2], O. S. ROATH[2], E. B. ROBERTS[2], J. STUART[2], G. M. TAYLOR[1], R. B. THOMPSON[2], G. WETHERLEY-MEIN[2], J. A. WHITTAKER[2] AND E. WILTSHAW[1,2]

Received 20 May 1977 Revised 2 September 1977 Accepted 7 September 1977

**Summary.—Seventy-one patients suffering from acute myeloid leukaemia (AML) who were already in complete remission and had already received one further course of cytotoxic drugs as consolidation therapy were randomised to receive maintenance chemotherapy alone or the same maintenance chemotherapy plus immunotherapy with BCG and irradiated allogeneic blast cells. The duration of first remission was slightly, but not significantly, longer in those patients who received immunotherapy. This was true also for the duration of survival after relapse. Comparison with other series suggested that the effect of such immunotherapy on duration of survival after relapse is probably real, but did not clearly indicate whether or not any real difference in the first remission duration existed.**

UNTIL 1972, the only clinical trial in which immunotherapy had been claimed to be beneficial in the treatment of any form of malignant disease in man was that of Mathé *et al.* (1969) in acute lymphoblastic leukaemia. However, larger, though not identical, trials carried out by the Medical Research Council's Working Party on Leukaemia in Childhood (MRC, 1971) and by the Leukemia Study Group A (Heyn *et al.*, 1975) failed to confirm Mathé's findings.

In 1969, a series of small studies was undertaken to assess the effects of immunotherapy on the duration of first remission, and on the survival, of adult patients suffering from acute myeloid leukaemia (AML) who were already in remission and who were all receiving regular chemotherapy as maintenance treatment. In these studies, new patients were allocated alternately to receive, if they later entered complete remission, maintenance treatment with chemotherapy only or with identical chemotherapy plus immunotherapy (Powles *et al.*, 1973). All the chemotherapy, both during induction and during remission, was administered at St. Bartholomew's Hospital, London (Bart's) but those patients who were allocated to receive immunotherapy were in addition seen regularly at the Royal Marsden Hospital, where all the immunotherapy was administered. By August 1972, these series were all quite small, but the duration of first remission and of survival were, overall, somewhat better in the immunotherapy group than in the controls. The studies were numbered 1, 2, 3, 4a and 4b, each containing one or

Reprints from the MRC Leukaemia Unit, Royal Postgraduate Medical School, London, W12

two dozen patients, and when Studies 2, 3 and 4a, all of which involved a similar form of immunotherapy, were pooled, the apparent effects of immunotherapy on duration of first remission and on survival were moderately significant statistically ($P<0{\cdot}05$). Although the immunotherapy was all administered at the Royal Marsden, this pool of Studies 2, 3 and 4a has come to be known as "the Bart's trial", and the chemotherapeutic regime used in Study 3 has come to be known as "Bart's 3 chemotherapy".

The encouraging preliminary results from the Bart's trial indicated the need for a randomised trial duplicating their study on a larger scale, and we now report the results of the trial organized by the Medical Research Council's (MRC) Working Party on Leukaemia in Adults which began in January 1973 with this purpose.

For practical reasons, the MRC trial was confined to Hammersmith Hospital and Oxford until 1974. In 1974, 6 hospitals in the Birmingham region joined the trial. At Manchester Royal Infirmary the research team had already had some experience with immunotherapy (Freeman *et al.*, 1973) and wanted to randomise their patients between immunotherapy alone and chemotherapy plus immunotherapy to discover whether chemotherapy during remission was of value to patients receiving immunotherapy. The Manchester part of the MRC study will be reported separately.

At Birmingham, Hammersmith and Oxford 148 patients presented; 77 of these achieved complete remission (52%) and 71 of these were randomised between chemotherapy and chemotherapy plus immunotherapy.

### PATIENTS AND METHODS

From 1st January, 1973 to 30th June, 1976, 71 patients who had entered complete remission were allocated at random to receive maintenance therapy, either with chemotherapy alone (C, 24 patients) or with immunotherapy as well as the same chemotherapy (C+I, 47 patients). The follow-up is complete to 1st July 1977.

The chemotherapy schedules for induction and maintenance therapy were identical with those described by Powles *et al.* (1973) except that doxorubicin was occasionally substituted for daunorubicin, at half the dosage, to reduce the risk of cardiotoxicity. For details of the induction chemotherapy with the 5-day course of daunorubicin and cytosine arabinoside repeated at 10-day intervals, and of the monthly maintenance chemotherapy courses of the same drugs, alternating with thioguanine and cytosine arabinoside, see Powles *et al.* (1973).

The *immunotherapy* involved the weekly injection of $\sim 10^9$ allogeneic irradiated blast cells (each patient receiving, as far as possible, cells from a single donor). The suspensions were injected intradermally (i.d.) and s.c. at 3 sites on the limbs (the volume being too great for purely i.d. injection). At a 4th site Glaxo freeze-dried BCG ($\sim 10^6$ organisms) was injected percutaneously to a depth of 2 mm through 40 punctures made with a Heaf gun. This regimen was identical with that used in Study 3 of the Bart's trial (Powles *et al.*, 1973). As in that trial, maintenance chemotherapy was discontinued after one year; Group C patients then received no further treatment until they relapsed, while Group (C+I) patients continued to receive immunotherapy but no chemotherapy. Patients who relapsed received the same treatment as was used to induce their first remission, but some who relapsed within 120 days, or who relapsed a second time, then received more intensive therapy. Patients in Group (C+I) whose disease entered remission a second time continued to receive immunotherapy as part of their maintenance therapy.

The MRC trial was designed to duplicate the Bart's trial in all respects and in the planning of the trial, and throughout its duration, close collaboration was maintained with the late Professor G. Hamilton Fairley and Dr R. L. Powles to ensure that the details of every procedure in the MRC trial were as nearly as possible the same as in the Bart's trial. Nevertheless, there were some minor differences in procedure between the two trials. These differences were as follows:

(1) In the Bart's trial, the allocation to maintenance chemotherapy only (C) or to chemotherapy and immunotherapy (C+I) was made to alternate patients at the time of first presentation, before remission had been induced. In the MRC trial, the allocation was made randomly, at the time when the patient was first recorded as having entered a state of complete remission. The randomization procedure was adjusted so that twice as many patients would be allocated to receive (C+I) as C.

(2) In the Bart's trial, immunotherapy was begun "whenever possible, just before complete remission, at a time when the marrow was hypoplastic" (Powles *et al.*, 1973). This was possible because the patients had already been allocated to receive immunotherapy. In the MRC trial, randomization was deferred until complete remission was established, as shown by the regeneration in the marrow of normal haemopoietic cells as well as the reduction of blast cells below 5%. After the first marrow sample considered to show complete remission, one further "consolidation" course of chemotherapy was given before starting immunotherapy. Thus, immunotherapy was begun 1 to 4 weeks later in the MRC trial patients than in the Bart's trial patients.

(3) In the MRC trial, the marrow was examined on the first day of every monthly course of maintenance chemotherapy, with the object of detecting relapse as early as possible. Blast-cell counts above 5% were considered to indicate relapse, but to allow for variation between different sites in the marrow, and for observer variability, a single sample of marrow showing 5–10% of blasts was not accepted as indicating relapse unless blast cells were present in the peripheral blood film at the same time. If they were not, a second marrow sample was taken 4 weeks later, and a blast-cell count above 5% was then accepted as indicating relapse. In the Bart's trial, marrow examinations were not made routinely during remission, being performed only when indicated by the peripheral blood and clinical findings.

(4) In the MRC trial, in accordance with the practice in earlier trials, patients aged 20 years and over who had undifferentiated leukaemia were eligible for entry, whereas in the Bart's trial they were deliberately excluded, entry being restricted to patients with leukaemia showing definite myeloid (granulocytic and/or monocytic) differentiation.

(5) Finally, in the Bart's trial, when patients relapsed, "the initial induction treatment with daunorubicin and cytosine arabinoside was repeated whenever possible". In the MRC trial, participants were encouraged to do this, but were free to use more intensive treatment for patients who relapsed within 120 days or for a second time.

### COMPLICATIONS OF IMMUNOTHERAPY

In general, immunotherapy was well tolerated; however, severe reactions occurred in a few cases. The sites of the BCG inoculations were sometimes slow to heal and, when persistently discharing ulceration occurred, inoculations of BCG were discontinued until the ulcers had healed. In 2 cases in which the BCG inoculation sites healed normally, the sites of the blast-cell injections became inflamed and ulcerated, the patients became febrile, and BCG organisms were recovered from the ulcer. These patients were considered to have developed systemic BCG disease and were successfully treated with antituberculosis drugs. Rarely, patients complained of generalised muscle aching and weakness during the 24 h following immunotherapy, and most patients complained of transient pain at the inoculation sites.

In 3 immunotherapy patients temporary iritis was reported, though no explicit search for this condition was organised.

### STATISTICAL METHODS

These are as described by Peto *et al.* (1976; 1977) and consist of Kaplan-Meier life tables and logrank *P* values*. For the latter, exact variance calculations were performed (*ibid.*, Statistical Note 7) and continuity corrections were not used (*ibid*, p. 38).

* Because we were testing a previously published claim that immunotherapy *helps*, we have cited one-tailed *P* values when comparing treatments, but otherwise we have cited ordinary *P* values.

One-tailed *P* values give the probabilities of observing, by chance alone, the differences we did observe *in favour* of immunotherapy. Obviously, if chance alone were operating, there would be an equal probability of observing an equivalent difference *in the opposite direction*, and the total probability of observing a difference of the magnitude we did, which is the ordinary *P* value, is therefore double the one-tailed *P* value which we cite when comparing treatments.

RESULTS

Data for each randomised patient are listed separately in the Appendix. We have examined 3 different measures of outcome:

(1) Duration of complete remission (all 71 patients: 24 C, 47 C+I).
(2) Duration of survival from the date of first relapse (analysed for the 60 patients who relapsed before 1 July 1977: 23 C, 37 C+I).
(3) Duration of survival from randomization (all 71 patients).

(1) *Duration of complete remission*

(*I.e.* time from definite remission to first relapse or, for 2 patients who died while still in their first remission, to death.)

There were no significant differences in the duration of first remission between patients of different ages or patients from different centres (Oxford, Hammersmith or Birmingham region) (Table I). Although the immunotherapy patients fared slightly better (Fig. 1) this difference was not statistically significant (Table I) (whether tested directly or after retrospective stratification for centre.) "Retrospective stratification for centre" (Peto *et al.*, 1976; 1977) implies that, in making the overall treatment comparison, patients at one centre are not assumed to be comparable with patients elsewhere.) The ratio of the relapse rate among immunotherapy patients to that among the controls was 0·74, but because the trial is not large, the 95% confidence interval for this ratio is rather wide (~0·45–1·20) and our data are thus consistent both with no effect whatsoever for immunotherapy and with a halving of the relapse rate by immunotherapy.

(2) *Duration of survival from the date of relapse*

The median survial after relapse had been diagnosed was only 124 days (18 weeks) and it tended to be shorter in the Birmingham area (14 weeks) than at Hammersmith and Oxford (22 weeks).

After relapse, the immunotherapy+chemotherapy patients (Fig. 2) lived somewhat longer (median 22 weeks) than the chemotherapy only patients (14 weeks). However, such medians are usually rather

FIG. 1.—Life-table estimate of the probability of survival in complete remission of 71 patients allocated at random for maintenance treatment by chemotherapy alone (C, 24 patients ——) or chemotherapy and immunotherapy (C+I, 47 patients – – – –). (All patients have been followed up for 18 or more months.) Solid symbols, relapse; open symbols, incomplete follow-up.

FIG. 2.—Life-table estimate of the probability of survival after the first relapse of 60 patients who relapsed before 1 July 1977. 37 patients were in the group receiving both chemotherapy and immunotherapy (– – – –) and 23 in the group receiving only chemotherapy (————). (All but 3 of these patients have been followed up for over a year, but 9 more may yet relapse and thus enter this comparison.) Solid symbols, death; open symbols, incomplete follow-up.

unreliable* because medians are derived from only a single point on the whole survival curve. It is, therefore, usually preferable to use a logrank test to see whether, taking the entire shapes of the two curves into consideration, the differences between the two curves can be plausibly attributed solely to chance. Unfortunately, as with remission duration, the results are equivocal. There is no statistically significant difference in survival after relapse (chi-square on one degree of freedom=2, one-tailed $P=0{\cdot}08$) yet the data are consistent not only with no effect but also with at least a halving of the death rate among relapsed patients (observed death rate ratio=0·68, 95% confidence interval ~0·40–1·15).

(3) *Survival from randomization* (i.e.) *from the onset of remission*)

As might be expected, the relationship of treatment to overall survival is similar to that with remission duration (Fig. 3 and Table III). Again, the data are consistent both with a halving of the death rate and with no effect whatever. (The observed death rate ratio was 0·67, with 95% confidence interval ~0·39–1·14.)

### DISCUSSION

The present trial was started in 1973, shortly after an unpublished statistical analysis of the Bart's immunotherapy trial suggested that the duration of remission was significantly longer if, during remission, the patients received weekly immunotherapy in addition to monthly courses of chemotherapy, instead of the same chemotherapy alone. Analysis of the Bart's results one year later (Powles *et al.*, 1973) suggested that, in addition to the prolongation of the first remission, the duration of survival after the first relapse and the overall survival were also superior in the group of patients who had received immunotherapy.

Further follow-up of the original Bart's trial (Powles *et al.*, 1977) has shown that the statistical significance of the effect on first remission which they originally reported (Powles *et al.*, 1973) no longer exists. Now that more complete information is available for all their patients, it appears that the effect reported earlier on the duration of first remission could have been a temporary artefact of chance, and all but one patient in their original immunotherapy group had relapsed by the end of 1976.

In our data, the immunotherapy-treated group has had slightly longer durations, both of first remission and of survival after first relapse. However, both

* For example, although the discrepancy of 8 weeks between these two medians looks promising, consider the medians we would have obtained if just one extra immunotherapy patient had relapsed. If this hypothetical patient had died 6 months after his relapse, the present medians would be unchanged, but if he had died *four* months after his relapse the discrepancy between the medians of the two treatment groups would be reduced to 4 weeks.

FIG. 3.—Life-table estimate of the probability of survival after randomization of 71 patients in complete remission who received either chemotherapy alone (C, 24 patients ———), or chemotherapy and immunotherapy (C+I, 47 patients – – – –). (All patients have been followed up for 18 or more months.) Solid symbols, death; open symbols, incomplete follow-up.

these differences (and the difference with respect to total survival) are easily compatible with (a) immunotherapy being completely without benefit, and (b) immunotherapy halving the relapse rate, the death rate among relapsed patients, or both.

A larger trial would have been much more informative, but this was not achieved for the reasons already described; but, it should be remembered, our trial is larger than any other randomized trial of immunotherapy for AML yet reported. To obtain independent confirmation or refutation of the original Bart's results, we must therefore look to other series of patients. Two questions need answering: Does immunotherapy prolong first remission? and does immunotherapy prolong survival after relapse?

*First Remission*

At a meeting at the National Cancer Institute (NCI, 1977) in October 1976, it was intended that all randomized trials of immunotherapy should be reviewed together.

Four other randomized AML trials were presented, in addition to the Bart's and MRC trials. The South Eastern Cancer Study Group reported $P<0{\cdot}05$ for the duration of first remission, but this difference appeared to exist more because their controls fared unduly badly (median 13 weeks to relapse) than because their immunotherapy patients (median 25 weeks) fared well, and may, therefore, be an artefact of chance. However, in all 6 trials (Bart's, MRC, S.E. U.S.A., S.W. U.S.A., South Wales and Central Sweden) first remissions were somewhat longer among immunotherapy patients, although in 5 of these 6 trials the differences were not statistically significant. This indicates, again inconclusively, that immunotherapy does prolong first remission.

Less reliable evidence may be derived by comparing what happened to single groups of AML patients at various different centres who achieved remission concurrently with the patients in the MRC trial. All other centres participating in the 6th AML trial during the period of intake to the present trial used, in one arm or other, the same chemotherapy and criteria of remission as in the present trial. All except Manchester used no immunotherapy and, among the 40 such patients who achieved remission, first remissions were actually slightly longer than among either group in our randomized trial. Conversely, 10 patients at Manchester received immunotherapy as well as this chemotherapy, and their first remissions were slightly shorter than in our randomized trial. Finally, 41 patients remitting at Bart's during this period (after closure of their trial) received various different forms of chemotherapy and various different forms of immunotherapy, and their first remissions were also slightly shorter than among either group in our randomized trial. These three concurrent comparisons are all uncertain, because the treatments were not allocated by randomization, but none suggests that immunotherapy prolongs first remissions.

*Survival After Relapse*

Here, the 5 other randomized studies and the 3 concurrent comparisons all indicate, as was originally suggested by Freeman *et al.* (1973) that immunotherapy does prolong survival after relapse. In all 6 randomized trials (NCI, 1977) survival after relapse was better for immunotherapy patients, and in 3 of these the differences were apparently statistically significant. (However, the detailed data from one of these 3 trials have been tabulated by Whittaker and Slater (1977) and re-analysis of the tabulated data indicates that although a difference certainly exists, it is not statistically significant.)

In the MRC 6th chemotherapy trial, 28 of the (already mentioned) 40 patients treated according to the "Bart's 3" protocol relapsed, and these 28 then died more quickly after relapse than either of the two groups in the present randomized trial. By contrast, both the group of 10 C+I patients who relapsed at Manchester, and the group of 31 C+I patients who presented at Bart's after closure of their trial and relapsed before 1977, lived longer after relapse than did either randomized group in the present trial. This reinforces the suggestion that immunotherapy can prolong survival after relapse, but again the possible biases inherent in such non-randomized comparisons should be emphasized.

*Other Reports of the Present Trial*

At the NCI review meeting in October 1976 (NCI, 1977) our results to the end of September 1976 were presented. The present paper reports our results 9 months later, when 16 further relapses and deaths have occurred (6 among C-only patients and 10 among C+I patients, because two-thirds of our patients were allocated to receive C+I). The present uncertain results are almost identical with the results presented then. However, at the MRC annual review meeting, a significant effect of immunotherapy in prolonging survival after relapse was reported from this trial. This was because all 6 of the C-only events occurred before 1 January 1977, while only 2 of the 10 C+I events did so. In small trials, quite rapid changes can always occur, and because 19 patients remain alive (9 in first remission) further fluctuation is still possible but perhaps unlikely.

### CONCLUSION

It seems probable, though not certain, that immunotherapy can prolong survival after relapse, and it is possible that it can slightly extend first remissions. The mechanism in either case remains obscure, and comparisons with successful immunotherapy in experimental animals are not necessarily relevant. If our "immunotherapy" really does have some effect, elucidation of its mechanism might lead to improvement in its efficacy. It is, however, not yet clear that future efforts to improve survival in AML should chiefly be directed to improving immunotherapy, and we have not included immunotherapy in the current (7th) MRC AML trial.

Several laboratory tests, including studies of the immunological responses of the patients receiving immunotherapy in the trial reported here, have been carried out, and several of the blast-cell preparations used for immunization proved to be immunogenically "inert" (personal communication: Dr I. C. M. MacLennan, Dr D. Gale, Dr R. Harris, Dr G. M. Taylor). It remains possible, therefore, that other forms of immunotherapy may be more effective. In experimental animals immunotherapy is effective only when the number of residual tumour cells is very small.

Possibly our immunotherapy was begun too soon or too late to be maximally effective. The Manchester group have suggested more intensive consolidation therapy before randomizing between immunotherapy and no other maintenance treatment, and they are now carrying out a trial so designed.

The principal physicians entering patients were Dr Goldman (Hammersmith), Dr Callender (Oxford) Dr Shinton (Coventry), Dr Giles (Stoke), Dr Pollock (Birmingham), Dr Stewart (Birmingham QE), Dr Bagshawe (Charing Cross) and Dr Allan (Wolverhampton).

Colleagues in radiotherapy and radiobiology irradiated the blast cells weekly before injection. The data were collected by Miss M. Gilham and analysed by Mr R. Peto and Dr H. Cuckle. Miss M. Gilham, Mrs D. L. Haysome, Miss A. Spira, Ms R. Coggins and Ms G. Mead typed protocols and manuscripts.

### REFERENCES

FREEMAN, C. B., HARRIS, R., GEARY, C. G., LEYLAND, M. J., MACIVER, J. E. & DELAMORE, I. W. (1973) Active Immunotherapy Used Alone for Maintenance of Patients with Acute Myeloid Leukaemia. *Br. med. J.*, iv, 571.

HEYN, R. M., JOO, P., KARON, M., NESBIT, M., SHORE, N., BRESLOW, N., WEINER, J., REED, A. & HAMMOND, D. (1975) B.C.G. in the Treatment of Acute Lymphoblastic Leukaemia. *Blood*, **46**, 431.

MATHÉ, G., AMIEL, J. L., SCHWARZENBERG, L., SCHNEIDER, M., CATTAN, A., SCHLUMBERGER, J. R., HAYAT, M. & DE VASSAL, F. (1969) Active Immunotherapy for Acute Lymphoblastic Leukaemia. *Lancet*, i, 697.

MEDICAL RESEARCH COUNCIL (1971) Treatment of Acute Lymphoblastic Leukaemia. *Br. med. J.*, iv, 189.

MEDICAL RESEARCH COUNCIL (1975) The Relationship between Morphology and Other Features of Acute Myeloid Leukaemia, and their Prognostic Significance. *Br. J. Haemat.*, **31** (Supplement), 165.

NATIONAL CANCER INSTITUTE (1977) *Immunotherapy of Cancer: Present Status of Trials in Man.* Proceedings of a meeting held on October 27–29, 1976. Ed. W. D. Terry and D. Windhorst. New York: Raven Press.

PETO, R., PIKE, M. C., ARMITAGE, P., BRESLOW, N. E., COX, D. R., HOWARD, S. V., MANTEL, N., MCPHERSON, K., PETO, J. & SMITH, P. G. (1976; 1977) Design and Analysis of Randomised Clinical Trials Requiring Prolonged Observation of Each Patient. *Br. J. Cancer*, **34**, 585; **35**, 1.

POWLES, R. L., CROWTHER, D., BATEMAN, C. J. T., BEARD, M. E. J., MCELWAIN, T. J., RUSSELL, J., LISTER, T. A., WHITEHOUSE, J. M. A., WRIGLEY, P. F. M., PIKE, M., ALEXANDER, P. & HAMILTON-FAIRLEY, G. (1973) Immunotherapy for Acute Myelogenous Leukaemia. *Br. J. Cancer*, **23**, 365.

POWLES, R. L., RUSSELL, J., OLIVER, T., WHITEHOUSE, J. M. A., CHAPRIS, B., MALPAS, J., CROWTHER, D. & ALEXANDER, P. (1977) Immunotherapy for Acute Myelogenous Leukaemia: Analysis of a Controlled Study 2½ Years after Entry of the Last Patient. *Br. J. Cancer*, **35**, 265.

WHITTAKER, J. A. & SLATER, A. J. (1977) The Immunotherapy of Acute Myelogenous Leukaemia using Intravenous B.C.G. *Br. J. Haemat.*, **35**, 263.

*Note added in proof.*—By 1 November 1977, all patients had been followed for 22 or more months, and on further statistical analysis the 3 treatment chi-squares (retrospectively stratified for centre) became 1·53 (first remission), 1·97 (survival after relapse) and 2·61 (overall survival). None of the explanatory variables listed in the Appendix (no. of courses, sex, age, morphology, centre or WBC) were significantly related to any measure of outcome, nor did retrospective stratification for any of them materially affect the relationships of treatment with outcome. (However, patients with only 3, 4 or 5 courses of tratment during the first 12 weeks after diagnosis did live slightly longer than those with 6, 7 or 8 courses, and the apparent effect of immunotherapy was somewhat greater among the latter than among the former patients.) Because the Bart's trial excluded patients with undifferentiated leukaemia, we finally re-analysed our data excluding our 9 such patients ; their prognosis was slightly worse than average, and no material change in any of our 3 treatment comparisons followed their exclusion.